# Characterization of Gelatin and Hydrolysates from Valorization of Farmed Salmon Skin By-Products

**DOI:** 10.3390/polym13162828

**Published:** 2021-08-23

**Authors:** José Antonio Vázquez, Carolina Hermida-Merino, Daniel Hermida-Merino, Manuel M. Piñeiro, Johan Johansen, Carmen G. Sotelo, Ricardo I. Pérez-Martín, Jesus Valcarcel

**Affiliations:** 1 Group of Recycling and Valorization of Waste Materials (REVAL), Instituto de Investigaciones Marinas (IIM-CSIC), C/Eduardo Cabello 6, CP36208 Vigo, Pontevedra, Spain; jvalcarcel@iim.csic.es; 2 CINBIO, Departamento de Física Aplicada, Facultad de Ciencias, Universidade de Vigo, CP36310 Vigo, Pontevedra, Spain; cahermida@uvigo.es (C.H.-M.); mmpineiro@uvigo.es (M.M.P.); 3 Netherlands Organization for Scientific Research (NWO), DUBBLE@ESRF, CS 40220, F38043 Grenoble, France; daniel.hermida_merino@esrf.fr; 4 Norwegian Institute of Bioeconomy (NIBIO), Torggården, Kudalsveien 6, NO-8027 Bodø, Norway; johan.johansen@nibio.no; 5 Group of Food Biochemistry, Instituto de Investigaciones Marinas (IIM-CSIC), C/Eduardo Cabello 6, CP36208 Vigo, Pontevedra, Spain; carmen@iim.csic.es (C.G.S.); ricardo@iim.csic.es (R.I.P.-M.)

**Keywords:** salmon (*Salmo salar*), gelatin, valorization, aquaculture by-products, gel strength, rheological properties, protein hydrolysates, absolute molecular weight

## Abstract

Salmon processing commonly involves the skinning of fish, generating by-products that need to be handled. Such skin residues may represent valuable raw materials from a valorization perspective, mainly due to their collagen content. With this approach, we propose in the present work the extraction of gelatin from farmed salmon and further valorization of the remaining residue through hydrolysis. Use of different chemical treatments prior to thermal extraction of gelatin results in a consistent yield of around 5%, but considerable differences in rheological properties. As expected from a cold-water species, salmon gelatin produces rather weak gels, ranging from 0 to 98 g Bloom. Nevertheless, the best performing gelatins show considerable structural integrity, assessed by gel permeation chromatography with light scattering detection for the first time on salmon gelatin. Finally, proteolysis of skin residues with Alcalase for 4 h maximizes digestibility and antihypertensive activity of the resulting hydrolysates, accompanied by the sharpest reduction in molecular weight and higher content of essential amino acids. These results indicate the possibility of tuning salmon gelatin properties through changes in chemical treatment conditions, and completing the valorization cycle through production of bioactive and nutritious hydrolysates.

## 1. Introduction

Gelatin is a peptide mixture resulting from the denaturation of collagen, the main structural protein in connective tissue. Collagen consists of a triple helix of peptides of around 100 kDa (α-chains), assembled in the extracellular matrix into aggregates forming fibrils [1]. Disruption of the triple helix structure turns very insoluble collagen into soluble gelatin, a more tractable material that has found many applications in the food, pharmaceutical, and biomedical industries [2,3,4].

Sources of commercial gelatin comprise mostly pig and cattle bones, skins, and hides, but growing interest exists in fish gelatin. On the one hand, as a substitute of terrestrial animals for cultural reasons, but also as properties of fish gelatins, different to those of mammalian counterparts, may better suit particular applications [2,3]. As a result, many works have dealt with the extraction and properties of gelatin from a wide range of fish species [5,6].

By-products of the fishing industry rich in collagen may turn into raw materials for gelatin extraction, especially skins which may contain four-fold more collagen than heads or bones [7]. This biomass is currently discarded or used to produce low-value fish feed, and may pose environmental problems as its volume expands along with growing consumption of fish, increasingly processed as modern societies demand convenience food, such as fish fillets. In this line, valorization of processing by-products from highly demanded species, such as salmon, is particularly relevant.

A number of previous studies have focused on the isolation of gelatin from salmon skin, looking at the effect of different pretreatments and thermal extraction conditions on yield and properties of the resulting gelatin. The treatments commonly applied involve an alkaline step followed by acid [8,9,10,11,12], although some authors have tried initial saline treatment [7], sometimes followed by alkaline medium and trypsin [13,14], or trypsin alone [14]. Generally, harsher treatments and high extraction temperatures increase yield but result in gelatin with weaker rheological properties, which seem to be influenced by the content of imino acids, hydrophopic amino acids, and molecular weight. Despite the importance of the latter, studies to date have only used relative methods to assess the *M*_w_ of each gelatin fraction, commonly electrophoresis, or capillary viscometry, techniques that only provide a mean value for the whole material. 

In the present work, we have extracted gelatin from farmed salmon skin using different methods, and studied for the first time the molecular weight distributions of each material by gel permeation chromatography (GPC) with light scattering detection, allowing for absolute molecular weight measurements. The rheological properties of gelatin are discussed in light of this information, along with amino acid profiling. Furthermore, we propose to complete the valorization of salmon skin with the production protein hydrolysates from the solids remaining from gelatin extraction.

## 2. Materials and Methods

### 2.1. Skin By-Products from Farmed Salmon

Fresh skin from farmed Atlantic salmon (Salmo salar) resulting from the industrial filleting of this species was kindly provided by Dr. Johan Johansen (Norwegian Institute of Bioeconomy Research, NIBIO, Bodø, Norway). Frozen salmon skins were sent from Norway to Spain and stored at −20 °C until use. Skins were cut in portions less than 5 × 5 cm, and 500 g of these fragments were processed per batch. In all cases, the first step was a water wash step for 30 min under orbital agitation (50 rpm) to eliminate the impurities present in the skin fragments.

### 2.2. Production of Salmon Gelatin

Different protocols for gelatin extraction were evaluated (Table 1, Figure S1, Supplementary Materials). The first two protocols (P1 and P2) included in the first stage three sequential chemical treatments [15], repeating alkaline extraction twice to extract as much fat as possible). Protocol 3 (P3) followed the recommendations previously reported [16,17], repeating the alkaline extraction three times. Protocols 4 and 5 (P4 and P5) were based on the thermal extraction of gelatin in acidic conditions [18]. In all protocols, agitation was carried out in an orbital shaker and water wash for 30 min (1:4 ratio) applied in between steps.

The elimination of chemical effluents in each procedure (alkaline and acidic effluents) was carried out by filtration (1000 µm). At completion of the different chemical processing steps, the gelatin solution was then thermally extracted at 45 °C on aqueous medium (1:2 ratio) for 16 h in protocols P1–P3. Purification of the gelatin solutions was carried out in all protocols by filtration (500 µm), active charcoal adsorption (at 1.5% *w/v* for 2 h), and centrifugation (15,000× *g*/20 min). The clean supernatants derived from this last procedure were finally oven-dried for 48–72 h to obtain solid gelatin. In all cases each protocol (P1–P5) was executed in duplicate.

### 2.3. Production of Collagen Hydrolysates

The skin remains generated after the thermal extraction of gelatins (SR) were mixed, separated into aliquots of 0.8 kg, ground, and then hydrolyzed by two commercial proteases: Alcalase 2.4 L (Novozymes, Nordisk, Bagsvaerd, Denmark), and Papain 6000 (Gygyc Biocon, Barcelona, Spain). Experiments of hydrolysis were performed following the optimal conditions previously defined [19,20] (Table 2). All hydrolyses were performed in a 5 L glass-reactor (pH-Stat system equipped with additional temperature, agitation, and reagents-addition control), mixing 0.8 kg of skin remains in 1 L of distilled water employing 5 M NaOH for pH-control and maintaining continuous agitation at 200 rpm. At the end of the enzymatic digestion process, non-hydrolyzed materials were removed by filtration, and the liquid fraction was centrifuged (15,000× *g* for 20 min) to separate oil and hydrolysates. These hydrolysates were immediately warmed (90 °C/15 min) for protease inactivation. The hydrolysis degree (*H*, as %) was calculated according to the pH-Stat method and mathematical equations previously described [21,22]. The Weibull equation was applied to predict the experimental kinetics of *H* [23]:

(1) $$H=H_{m}\left\{1-\exp\left[-\ln2{\left(\frac{t}{\tau }\right)}^{\beta }\right]\right\}\;\mathrm{with}\;v_{m}=\frac{\beta H_{m}\ln2}{2\tau }$$

where *H* is the hydrolysis degree (%), *t* is the hydrolysis time (min), *H_m_* is the maximum hydrolysis degree (%), *β* is a dimensionless parameter associated with the slope of the hydrolysis process, *v_m_* is the maximum hydrolysis rate (% min^−1^), and *τ* is the time needed to reach the semi-maximum hydrolysis degree (min). The yield of digestion (Y_dig_) of raw material to the liquid phase was also calculated (in %) [23].

### 2.4. Chemical and Rheological Characterization

#### 2.4.1. Production Yields

Yields were calculated as the dry weight of gelatin extracted ×100 per wet weight of fresh skins before processing.

#### 2.4.2. Chemical Composition and Bioactive Properties

The chemical composition of salmon skin and gelatin was obtained by quantifying: (1) moisture, organic matter, and ash percentage [24], (2) total protein as total nitrogen × 6.11 [15,25], (3) total lipids [26], and (4) amino acid content by ninhydrin reaction [27] employing an amino acid analyzer (Biochrom 30 series, Biochrom Ltd., Cambridge, UK) and norleucin as internal standard. Regarding characterization of skin waste hydrolysates, the following was determined: (1) total soluble protein by the Lowry method [28], (2) in vitro digestibility by the Association of Official Agricultural Chemists (AOAC) official method according to the reformulations suggested by [25], and (3) in vitro antihypertensive activity Angiotensin I-converting enzyme (ACE) inhibitory activity (I_ACE_) calculating IC_50_ values (protein-hydrolysate concentration that generates a 50% of I_ACE_) by dose-response modeling [29,30]. All analyses were performed in at least duplicate.

#### 2.4.3. Molecular Weight

The molecular weight profiles of salmon gelatins were analyzed by gel permeation chromatography with an Agilent 1260 liquid chromatography (LC) system consisting of quaternary pump (G1311B), injector (G1329B), column oven (G1316A), DAD (G1315C) refractive index (G1362A), and dual angle static light scattering (G7800A) detectors. Proteema precolumn (5 µm, 8 × 50 mm), Proteema 100 Å (5 µm, 8 × 300 mm), Proteema 300 Å (5 µm, 8 × 300 mm), and Proteema 1000 Å (5 µm, 8 × 300 mm) (PSS, Mainz, Germany) were used for polymer separation. The system was kept at 20 °C and 0.15 M sodium acetate: 0.2 M acetic acid, pH 4.5 was used as mobile phase, at a rate of 0.5 mL/min. Samples were dissolved at 1.8–2.2 g/L in the GPC mobile phase. To avoid errors due to incomplete dissolution of samples, a refractive index increment (dn/dc) of 0.190 [31] was used to estimate the molecular weight of gelatin, and a dn/dc of 0.185 for the hydrolysates [32].

#### 2.4.4. Gel Strength

The strength of salmon gelatin was measured by the method detailed in [33]. Briefly, solutions of gelatin were prepared at a concentration of 6.67% (*w/v*), completely dissolved at 45 °C and cooled at 4 °C for 16–18 h [16]. Gel strength was measured using a Stevens-LFRA Texture Analyzer (Hucoa Erlöss S.A., Madrid, Spain) with a 1000 g load cell equipped with a 0.5 inch of diameter Teflon probe. A trigger force of 5 g and a penetration speed of 1 mm/s were used, and gel strength was expressed as maximum force (in g), taken when the plunger had penetrated 3 mm into the gelatin gels, as an average of three determinations.

#### 2.4.5. Infrared Spectroscopy

Fourier transformed infrared spectroscopy by attenuated total reflectance (ATR-FTIR, Thermo Fisher Scientific, Waltham, MA, USA) was obtained using a Spectrometer Nicolet 6700, equipped with a source IR-Turbo fitted with a detector based on deuterated triglycine sulfate (DTGS) in a beamsplitter of KBr. Background scans were 34 with a spectral resolution of 4 cm^−1^ at ambient temperature, using an Attenuated Total Reflectance (ATR) accessory. Gelatin was deposited in a gold support in a humid chamber to avoid evaporation during measurements. A study of the second derivative of the spectra of Amide I was carried out, using the first difference derivative (FDD) method.

#### 2.4.6. Thermogravimetric Analysis

Thermogravimetric Analysis (TGA) measurements were performed with a Setsys Evolution 1750 Simultaneous Thermogravimetric Analysis (TGA)/Differential Scanning Calorimetry (DSC) instrument (Setaram), presented in previous publications [34]. About 8.5 mg of samples were introduced in a sealed capsule, undergoing temperature sweeps from room temperature to 800 °C at a heating rate of 5 °C/min^−1^ under an inert nitrogen atmosphere to avoid oxidation.

#### 2.4.7. Rheological Properties

Rheological properties of the gelatin hydrogels were determined using a Physica MCR 101 Rheometer (Anton Paar, Graz, Austria), equipped with a cone-plate geometry (CP50-1), with a constant gap of 0.048 mm, used for strain sweep and frequency sweep measurements, and rugged plate-plate (PP50/S) with a gap of 0.1 mm, for measurements of temperature ramps, allowing to control torques between 0.5 mN·m and 125 mN·m [35]. The linear viscoelastic range was determined by performing a strain sweep from 0.001 to 1000% at a constant angular frequency of 10 rad/s for 30% by weight of the gelatin hydrogel. The storage modulus G′ and loss modulus G″ were determined in the range of linear deformation. Frequency sweep measurements were also made from 0.05 to 600 rad/s applying a constant 0.1% strain. All experiments were carried out at 20 °C.

### 2.5. Numerical Fittings and Statistical Analyses

Fitting procedures and parametric estimations calculated from the hydrolysis kinetics were carried out by minimizing the sum of quadratic differences between the observed and model-predicted values, using the non-linear least-squares (quasi-Newton) method provided by the macro ‘Solver’ of the Microsoft Excel software. Confidence intervals from the parametric estimates (Student’s *t* test) and consistence of mathematical models (Fisher’s *F* test) were evaluated by “SolverAid” macro (Levie’s Excellaneous website: http://www.bowdoin.edu/~rdelevie/excellaneous). The significance of comparisons between protocols were analyzed by analysis of variance (ANOVA) with a significance level of *p* < 0.05.

## 3. Results

### 3.1. Gelatin Extraction by Different Procedures

In the present work, we evaluated different chemical protocols performed at two temperatures, for the recovery of gelatin from the skin of Atlantic salmon. The yields of production were similar in all cases (around 5% *w/w*), not finding significant differences between protocols (*p* > 0.05) (Table 3). However, gel strengths were significantly higher employing P1 and P5, without differences between them, in comparison with the rest of protocols (*p* < 0.05). In fact, gelatins in P3 did not form gels.

The content of amino acids was also determined for all gelatins (Table 4). The major components were, in this order, glycine, glutamic acid and proline (in similar levels), alanine, arginine, and hydroxyproline. Regarding imino acids, hydroxyproline concentration was similar in all gelatins, except in P3 in which it was lower, whereas proline was significantly higher in P2 and P5. Taking into consideration the sum of both amino acids, gelatins from P1, P2, and P5 contain more than 18% (18.89% in P2) and around 17.5% in P3 and P4. The values of total protein present in dry gelatin from P1 and P2 were superior to 90%, without significant differences with P5 (*p* > 0.05). The presence of fat in P3 and P4 was higher than 10% (data not shown). In nutritional terms, the content in essential amino acids was similar for all chemical treatments of skins (ranging 26–28%).

### 3.2. Molecular Weight

**Figure 1:**
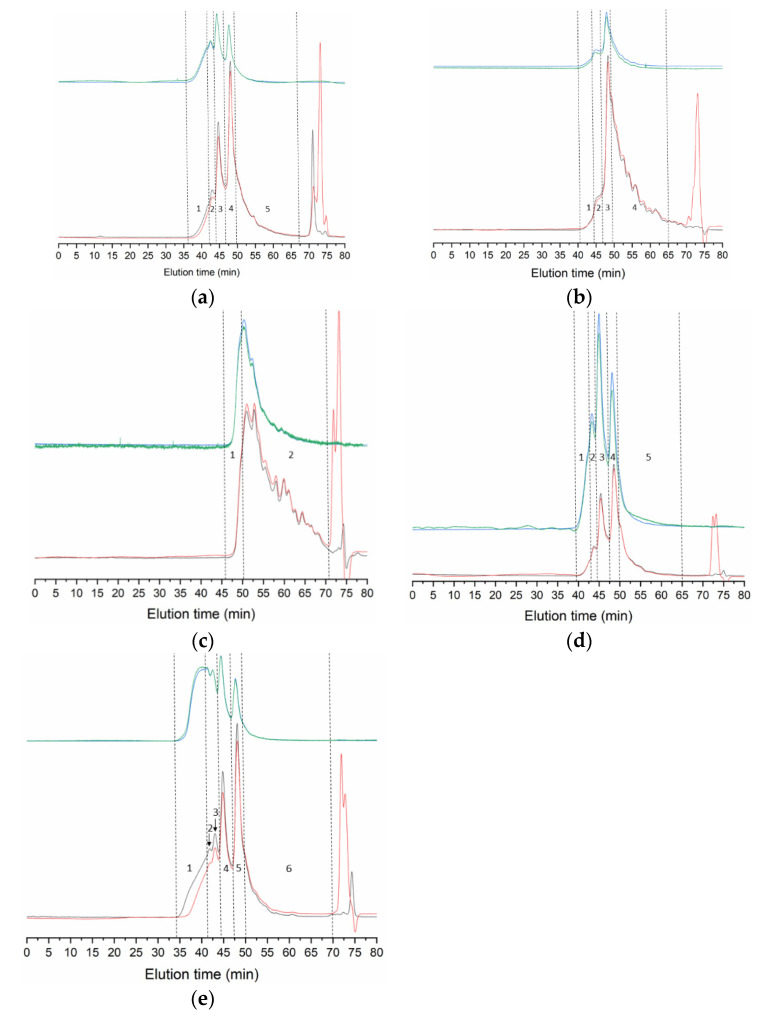
GPC eluograms of gelatin extracted from the skin of salmon by different methods: P1 (**a**), P2 (**b**), P3 (**c**), P4 (**d**), and P5 (**e**). Blue line: right angle light scattering; green line: low angle light scattering; red line: refractive index; and black line: ultraviolet (232 nm). Details of the numbered peaks/regions can be found in Table 5.

Profiles of gelatin samples analyzed by GPC (Figure 1) show two distinct patterns. In all samples except P3, the bulk of the polymeric material elutes early, approximately up to 49 min. Characteristic peaks appear at 43–44, 45–46, and 48–49 min in all three samples, corresponding to molecular weights of 312–352, 188–220, and 110–122 kDa (Table 5). The molecular weight of larger species could not be accurately determined, except for a small fraction in P5 of 482 kDa. These high *M*_w_ species account for 10.5% in P1, 1.5% in P4, and 16.4% in P5 of the total area, measured by the refractive index detector (RID).

Peptides of molecular weight below 100 kDa represent only 35.7%, 32.7%, and 22.0% of the polymeric material (RID area) in P1, P4, and P5, respectively, but account for more than 60% in P2 and 90% in P3. This is a heterogeneous fraction composed of multitude of overlapping peaks. In P3, elution starts before 49 min, and therefore we integrated a small fraction of 6.0% of the total area. Even though no clear peak appears, the tentatively estimated molecular weight reaches 105–112 kDa, close to the values obtained for the rest of the samples at equivalent elution times.

### 3.3. Thermal Stability

**Figure 2:**
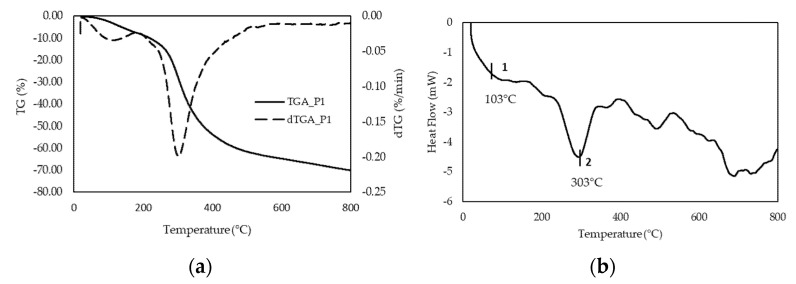
TGA thermogram (solid line, weight loss), and dTGA thermogram (dashed line, differential weight loss) obtained from TG curves (**a**); and DSC thermogram (**b**) of salmon gelatin P1.

We selected sample P1 for further study, based on the results of gel strength and molecular integrity determined by GPC. Thermogravimetric analyses were conducted in the range of room temperature to 800 °C to estimate the thermal stability of gelatin P1. The TGA thermogram in Figure 2a shows a profile of weight loss with increasing temperature that can be divided into two separate regions. First, a slight degradation step with onset at around 100 °C, this first stage leads to a weight variation of around −8%, due to loss of absorbed water (Table S1, Supplementary Materials) [36]. Second, a much more pronounced decline in mass close to 300 °C, carrying a 60% weight reduction. This stage corresponds to the degradation of the low molecular weight protein fraction, as well as structurally bound water [37]. The weight derivative confirms these observations as it shows a sharp peak with a maximum at around 100 °C, and a much more pronounced peak, with a maximum of 300 °C, as shown in Figure 2.

The DSC thermogram obtained from the analysis of gelatin P1 is displayed in Figure 2b. The thermogram shows an endothermic peak corresponding to the glass transition temperature (*T*_g_) at approximately 87 °C, followed by an endothermic with a heat of fusion reaching 286.97 J/kg (Table S2, Supplementary Materials). 

### 3.4. Infrared Spectroscopy

**Figure 3:**
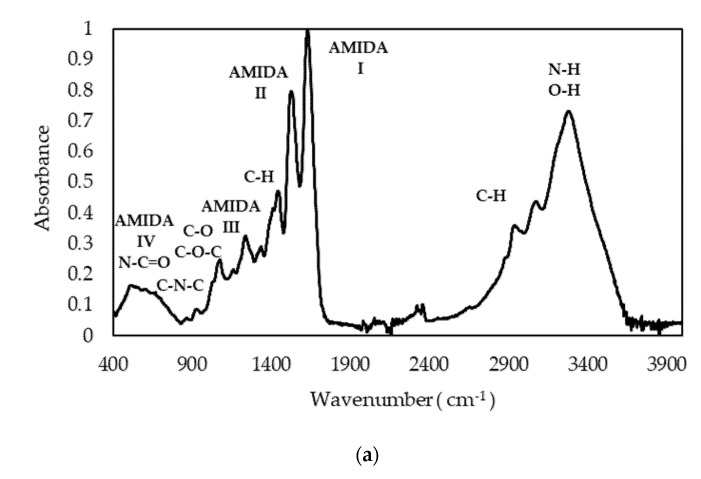

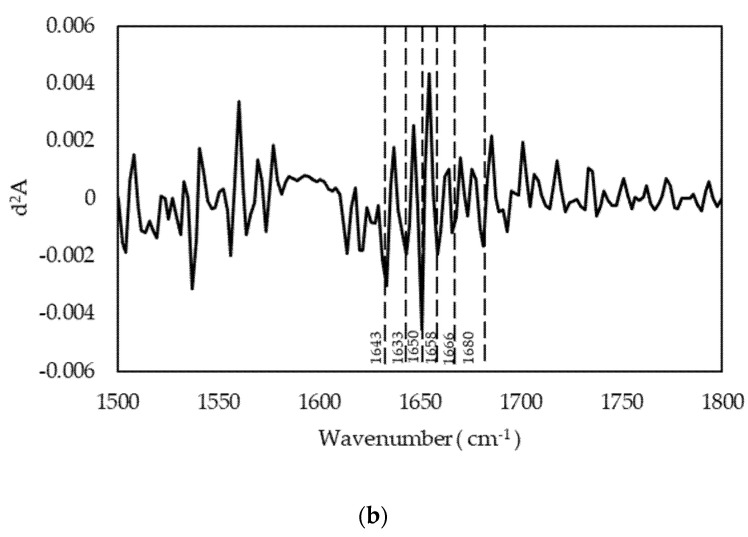
FTIR spectra of salmon gelatin P1 (**a**), and spectra of the second derivative (differential FTIR spectra, with FDD method) in the absorption region of Amide I (**b**).

The infrared spectrum of gelatin as a protein is characterized by the presence of several major absorption bands corresponding to vibrational transitions in the peptide chain. The FTIR spectrum shown in Figure 3a shows the characteristic gelatin bands. At high wave numbers, a broad and intense band appears with maxima at 3277 cm^−1^, due to the N–H bond tension modes of protein and O–H groups of carbohydrates and water. The signals between 2850 and 3000 cm^−1^ correspond to the tension modes of the C–H bonds of aliphatic chains, with bending modes emerging at 1334 and 1444 cm^−1^. The 1632, 1520, and 1237 cm^−1^ bands are due to the Amide I, II, and III bands, respectively. Other less intense bands emerging between 518, 600, and 700 cm^−1^ are due to Amide bands IV, V and VI, respectively. The rounded and broad shape of the band around 600 cm^−1^ is due to the presence of water in the sample. At 973 cm^−1^, a weak band appears corresponding to the symmetric tension mode of the CNC bond.

The Amide I was resolved into six components clearly seen on the second derivative spectra (Figure 3b), and compared with data from the reference literature [38]. The components’ peaks of the band span from 1620 to 1680 cm^−1^. The Amide I component at 1658 cm^−1^ is characteristic of a three-fold α-helix collagen [39]; components at 1633 cm^−1^ and 1643 cm^−1^ are often attributed to a CO group of imine type involved in hydrogen-bonding with H_2_O with some contribution from the *β*-sheets; and a –COOH band at 1680 cm^−1^ is assigned to the *β*-sheets with some contribution from the *β*-turn absorbance (Table 6). 

### 3.5. Viscoelastic Properties

**Figure 4:**
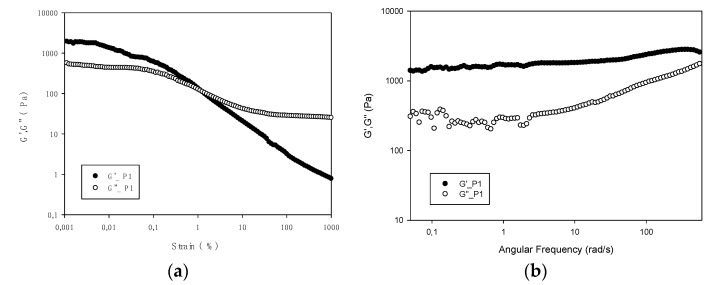
Strain and frequency sweeps for 30% solution (*w/v*) of salmon gelatin P1. Store (G′; filled symbol) and loss moduli (G″; hollow symbol) depicted versus strain (**a**) and angular frequency (**b**).

Initial strain sweeps were carried out at a frequency of 10 Hz to determine a suitable strain within the linear viscoelastic region for frequency sweeps. The strain sweeps allow the evaluation of the viscoelastic behavior of gelatins by determining the range where their rheological properties are independent of the applied deformation. Strain sweeps of a hydrogel prepared at 30% (*w/v*) with salmon gelatin P1 (Figure 4a) show broadly typical gel behavior, with a small plateau region in the moduli values with low strain value, and finally, significant drops in the moduli values and a transition to viscous dominant behavior.

The frequency sweep (Figure 4b) of salmon gelatin at a constant 0.1% strain shows that the elastic modulus G′ is greater than the viscous modulus G″ in the entire frequency range accessed [12] and both modules show a dependence of similar frequency [40]. A parallel storage and loss modulus profile of the frequency sweep from 0.05 rad/s to 10, flowing homogeneously confirming the gel network. At high frequencies there is a large increase in G″, which indicates a greater resistance of the gel [41].

**Figure 5:**
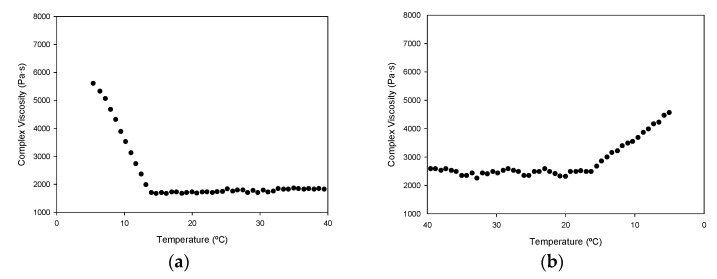

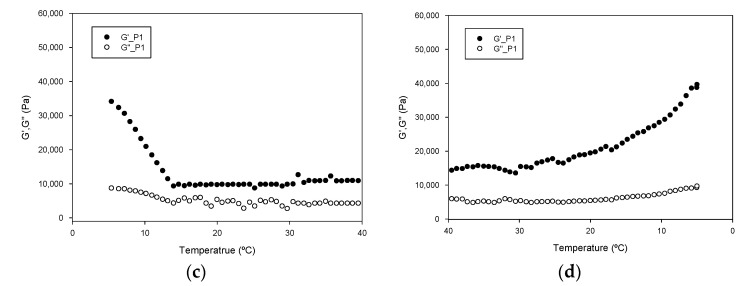
Variation with temperature of complex viscosity (**a**,**b**); and storage modulus (G′, filled circle) and loss modulus (G″, empty circle) (**c**,**d**) for a 30% solution (*w/v*) of salmon gelatin P1. Heating ramp (left column) from 5 to 40 °C, cooling ramp (right column) from 40 to 5 °C.

Rheological measurements of gelatin solution upon heating and cooling provide information about gelling kinetics, namely complex viscosity, storage, and loss moduli (Figure 5). As can be seen in (Figure 5a), a transition occurs around 14 °C in the complex viscosity of P1 (Figure 5a,b) [12]. In the heating ramp, a rapid decrease in complex viscosity, having less pronounced cooling, is followed by linear behavior, suggesting that the network structure formed during gelatinization was disrupted upon temperature increase. 

The values of the storage modulus G′ were found higher than the loss modulus G″ (Figure 5c,d) for P1 gelatin, indicating that the elastic behavior of the system is greater than the viscous behavior, forming of a large elastic network. Storage modulus with temperature follows the same pattern as complex viscosity. On the heating ramp, it shows a decrease in both modules to 14 °C (Figure 5c), which represents the transition from the gel state to the solution. On the cooling ramp, it produces a rather less pronounced peak for G′/G″ (Figure 5d). The increase in G′ in the cooling process is related to the transition from solution to gel state caused by triple helix formation. 

### 3.6. Production of Hydrolysates from Gelatin Extraction Skin-Waste

**Figure 6:**
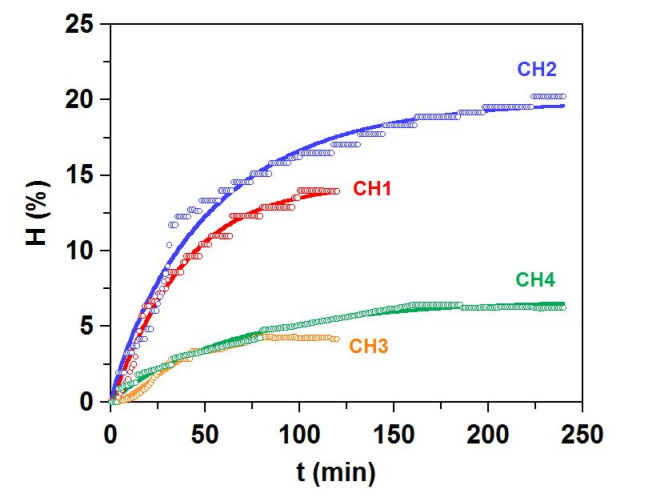
Proteolysis kinetics of salmon skins residues by alcalase (CH1 and CH2) and papain (CH3 and CH4). Experimental data of hydrolysis degree (H, symbols) were fitted to the Weibull equation (continuous line).

The rests of skin remaining from the thermal extraction of gelatin were hydrolyzed by two commercial endoproteases (alcalase and papain) at two times of proteolysis in both cases. The composition of those rests of skin was: 78.9 ± 6.8%, 19.5 ± 2.1%, 1.6 ± 0.4%, 29.4 ± 3.8%, and 68.5 ± 3.2%, for wet, organic matter, ash, total lipids, and total protein, respectively. Experimental data of hydrolysis degree, together with the trends modelled by Weibull equation, are represented in Figure 6. The statistical agreement between experimental and theoretical data was high (correlation coefficients ranging 0.979–0.987) and the consistency of fittings was recognized by means of Fisher’s *F* test (*p* < 0.005, data not shown). 

At longer skin proteolysis, the maximum degrees of hydrolysis (*H_m_*) were significantly larger (*p* < 0.05) and alcalase was clearly more efficient (values of *H_m_* and *v_m_*) than papain to digest skin residues (Table 7). Other parameters confirmed these results, namely higher yields of digestion, lower non-digestible skins recovered, and larger total protein content in the hydrolysates. As in gelatins, the most abundant amino acids in these materials were Gly and Glu (Table 7); however, glycine content in gelatin was higher than in the hydrolysates.

Furthermore, the percentage of imino acids (proline and hydroxyproline) was, in all situations, inferior to those showed in gelatins, i.e., CH3, the hydrolysate with the highest values of both compounds. The content in essential amino acids was higher for CH2 and CH1 and, in the four hydrolysates, these ratios were higher than those previously reported in salmon gelatins. On the other hand, the in vitro bioactivities of hydrolysates were influenced by the protease employed and by the time of hydrolysis. CH2 (alcalase for 4 h) showed significant improvements in both bioactivities, that is, the largest digestibility percentage, highest ACE-inhibition percentage and lowest IC_50_ value. The other alcalase hydrolysate, CH1, was the second most bioactive product at significant distance from the results found for papain hydrolysates. It was evident that the bioactive capacity of peptides from hydrolysates was directly dependent on the degree of hydrolysis (Table 7).

The molecular weight of hydrolysates produced with alcalase clearly differ from those digested with papain, as shown by the different elution profiles depicted in Figure S2 (Supplementary Materials). In the first case, elution starts around 60 min, while this occurs 10 min earlier in papain samples, indicating a significant difference in *M*_w_. Furthermore, the profile is more complex in CH3 and CH4, with a number of overlapping peaks, especially at shorter retention times.

The estimated molecular weights reflect the differences seen in elution profiles (Table 8). Samples hydrolyzed with alcalase resulted in a 4-fold reduction in *M*_w_ compared to those treated with papain, around 2 kDa in the first case and 8 kDa in the second. This was accompanied by more polydisperse distributions in papain hydroysates. Longer reaction times led to a reduction in *M*_w_ for both enzymes, although the effect was larger in alcalase hydrolysates.

## 4. Discussion

All protocols yield comparable amounts of gelatin (4.51–5.07%, Table 3), despite the differences in the chemical treatments and associated temperatures. This is unexpected, as previous studies have found significant effects of acid concentration, pretreatment temperature, and extraction temperature on gelatin yield from salmon skin [9,13]. The yield values obtained here agree well with a previous work reporting 5.5% yield (estimated based on a 15.3% yield in the skin separated from the muscle remaining in the salmon filleting by-product, with 35.7% skin and 64.3% muscle) [8]. Other works calculate yield based on hydroxyproline content, making comparison with the present data difficult. Furthermore, yield figures vary widely from 22.4% to 74% [7,9,13,14].

On the other hand, differences in chemical treatment do affect the gel strength of the resulting gelatins, to the point that P3 does not gel at all, while P1 and P5 reach similar values (98 and 92.5, respectively, Table 3). These results align with others previously reported [8,11,12]. Of note, treatment temperature shows opposite effects in P1 and P2 compared to P4 and P5. The latter only differ in the temperature of the alkaline treatment, which may indicate that low temperature enhances gel strength. If this holds true for P1 and P2, then the positive effect of risen temperature in the acidic treatments possibly compensates for the deleterious effect on NaOH treatment, as temperature remains constant throughout all three steps (NaOH, H_2_SO_4_, and citric acid). However, the higher gel strength seen after alkaline treatment at 4 °C, and acidic treatments at 22 °C cannot be generalized in light of the null gel strength of P3 (NaCl and NaOH at 4 °C, and acetic acid at 22 °C). 

Processing conditions show scant impact on the aminoacid profile of gelatin (Table 4). The main aminoacids in collagen (glycine, proline, and hydroproline) varied little across treatments, and while differences in proline content are statistically significant, the values range only from 10.20% to 11.39%. Although P3, which did not gel, contains the lowest amount of proline and protein content, no relationship with gel strength is apparent for the other samples. Hence, aminoacid composition does not explain differences in gel strength.

Molecular weight distributions, however, appear influenced by the process (Figure 1, Table 5), and related to differences seen in gel strength. In the strongest gel forming gelatins (P1 and P5), structural integrity is evidenced by distinct peaks of estimated *M*_w_ slightly above 300 kDa, 200 kDa, and 100 kDa, corresponding to the structural units of collagen (*γ*-, *β*-, and *α*-chains, respectively). Higher *M*_w_ species are due to supramolecular aggregates of the former. On the other hand, the only gelatin not capable of gelling (P5) only contains 6% of α-chains, the bulk of the material made of peptides below 100 kDa. These possibly consist of collagen fragments and aminoacids resulting from its degradation, and other non-collagenous proteins. Beyond these extreme cases, we failed to find a clear relationship between gel strength and the proportion of each structural unit. The behavior of intermediate strength gelatins illustrates this fact: while gel strength was slightly superior in P2 (53.5) than in P4 (44.5), around two thirds of P2 consist of protein below 100 kDa, whereas in P4 this fraction accounts for only one third. 

Based on the above results, we selected P1 as the preferred process to recover gelatin from salmon skin, and focused on this material for further characterization. As a starting point, we were interested in assessing the thermal stability of the gelatin sample. Weight loss determined by TGA (Figure 2a) behaved fairly similarly to other gelatins [42]. Furthermore, the high glass transition temperature determined by DSC (Figure 2b) suggests that the chain interactions are strong; this transition is related to the *M*_w_ and the chain architecture. *T*_g_ is a non-kinetic event, related to the mobility of the chains; therefore, depending on the solvent used, it may have a different Tg value, as reported in the literature [42]. Moreover, in DSC an endothermic peak appears around 300 °C, which corroborates the degradation of the low molecular weight protein fraction, as well as structurally bound water, which is observed in the thermogravimetric analysis.

The spectral FTIR profiles of gelatin sample P1 (Figure 3a) are typical of fish gelatin and comparable to others previously reported for other fish species [43]. We focused in particular in the Amide I band, as it is more sensitive to changes in the secondary structure. Qualitatively, the Amide I band can be explained by the superposition of bands corresponding to different conformational states of the polypeptide chain [43]. By applying the second derivative method (Figure 3b), we identified components (Table 6) assigned to the triple-helix conformation typical of tropocollagen, in agreement with the GPC elution profile. Furthermore, the presence of a –COOH band confirms that gelatin gelling is responsible for the situation wherein a certain number of carboxylic acid functions are blocked to form *β*-sheets and *β*-turns, thus forming a supramolecular network. The most intense signal corresponds to random coil disposition, indicating the predominance of a disordered structure. This may indicate that not only the peptides below 100 kDa (38.5%) contribute to this signal, but as do collagen structural units such as α-chains (28%), as determined by GPC (Table 5).

The rheological measurements of a hydrogel formed with gelatin P1 show typical gel behavior, which disappears as gelatin is heated above 14 °C (Figure 5). At this point, the network structure formed during gelatinization is disrupted. Previous works have reported similar values (15 °C) [12], but also lower (10–11 °C) [8], differences that may be influenced by the diverse extraction procedures used. 

The data presented here portrays gelatin as a viable option to add value to skin by-products resulting from fish filleting. However, the extracted gelatin only represents around 5% of the initial biomass (Table 3), leaving the bulk of the material as waste. To complete the valorization cycle, we propose the proteolysis of these skin remains. In the best case tested, Alcalase induces almost complete conversion of the substrate, leaving only around 5% of undigested material (Table 7). Concomitantly, the process manages to recover a significant amount of fish oil (up to almost 6%).

The hydrolysates still contain some collagenous material, as shown by the presence of proline and hydroxyproline (almost exclusive to collagen). The contribution of other proteins raises the ratio of essential aminoacids to 41.3%, comparable to hydrolysates prepared from other species [44,45,46], with digestibilities of up to 85.9%. Furthermore, in vitro antihypertensive activity in the Alcalase hydrolysate CH2 (82.3% inhibition; 71.2 µg protein/mL-IC_50_) compares favorably with hydrolysates from salmon heads (71.9% inhibition; 478.5 µg protein/mL-IC_50_) and frames and trimmings (87.0% inhibition; 653.7 µg protein/mL-IC_50_) [20]. These properties pose the hydrolysates as potential ingredients of food supplements, aquaculture feed, pet food, and microbial culture media.

Digestibility and antihypertensive activity (Table 7) appear correlated with the *M*_w_ of the hydrolysates, with more fragmented protein possessing higher digestibility and antihypertensive activity. However, previous studies have reported contradictory results, finding direct [47], inverse [48], or no relationship [20,32].

## 5. Conclusions

Extraction of gelatin from salmon skin and the production of hydrolysates from the remaining skin residues represent a viable path to recover protein from filleting by-products. Of all the methods tested, chemical treatment of the skin with sodium hydroxide, sulfuric and citric acids at 22 °C produces gelatin with the strongest rheological properties. These properties seem to correlate with a higher proportion of high molecular weight components and lower amounts of peptides below 100 kDa, but we found no relationship with amino acid composition. The molecular weight distributions seem to reflect in the bands seen in infrared spectroscopy, which are related to secondary structuring and random coil conformation of protein chains. Hydrolysis of the gelatin extraction by-products achieves higher efficiency with Alcalase, liquefying close to 80% of the initial solids into soluble low molecular weight peptides. Aminoacid composition, digestibility and antihypertensive activity of the resulting hydrolysates show their potential as food and feed ingredients.

## Figures and Tables

**Table 1:** Chemical treatments applied for gelatin extraction in each protocol. S:L solid to liquid ratio.

Protocol	Treatment 1	Treatment 2	Treatment 3
P1	0.05 M NaOH	0.02 M H_2_SO_4_	0.052 M citric acid
	S:L 1:4	S:L 1:4	S:L 1:4
	30 min	30 min	30 min
	50 rpm	50 rpm	50 rpm
	22 °C	22 °C	22 °C
P2	0.05 M NaOH	0.02 M H_2_SO_4_	0.052 M citric acid
	S:L 1:4	S:L 1:4	S:L 1:4
	30 min	30 min	30 min
	50 rpm	50 rpm	50 rpm
	4 °C	4 °C	4 °C
P3	0.8 M NaCl	0.2 M NaOH	0.05 M acetic acid
	S:L 1:4	S:L 1:6	S:L 1:10
	30 min	30 min	3 h
	50 rpm	50 rpm	50 rpm
	4 °C	4 °C	22 °C
P4	0.1 M NaOH	H_3_PO_4_ until pH 5–5.2	
	S:L 1:2	S:L 1:2	
	30 min	3 h	
	50 rpm	50 rpm	
	22°C	22 °C	
P5	0.1 M NaOH	H_3_PO_4_ until pH5–5.2	
	S:L 1:2	S:L 1:2	
	30 min	3 h	
	50 rpm	50 rpm	
	22 °C	22 °C	

**Table 2:** Experimental conditions for hydrolysis of skin remains after thermal extraction of gelatin.

Protocol	Enzyme	Temperature (°C)	pH	Time (h)
CH1	Alcalase 0.2% (*v/w*)	56.2	8.27	2
CH2	Alcalase 0.2% (*v/w*)	60.3	8.82	4
CH3	Papain 0.2% (*v/w*)	65	7.00	2
CH4	Papain 0.2% (*v/w*)	65	7.00	4

**Table 3:** Yield and gel strength for gelatin extracted from salmon fresh skins under the various protocols of production tested. Values are average ± intervals of confidence for *n* = 2 (replicates of independent batches) and α = 0.05. Different letters (a, b and c) in each file (as superscript) mean significant difference between protocols (*p* < 0.05).

Protocol	Yield (%, w of Gelatin/w of Skin)	Gel Strength (Bloom, g)
P1	4.73 ± 0.81 ^a^	98.0 ± 9.8 ^a^
P2	4.60 ± 0.66 ^a^	53.5 ± 1.0 ^b^
P3	5.07 ± 0.40 ^a^	0.0
P4	4.55 ± 0.36 ^a^	44.5 ± 2.9 ^c^
P5	4.79 ± 1.05 ^a^	92.5 ± 4.9 ^a^

**Table 4:** Amino acid (AA) content of gelatins recovered from fresh salmon skins (% or g/100 g total amino acids) with each protocol of production. OHPro: hydroxyproline. Pr: % of protein present, as the sum of amino acids, in the extracted gelatin sample, and TE/TA: ratio total essential amino acids for human/total amino acids. Errors are the confidence intervals for *n* = 2 (replicates of independent batches) and α = 0.05. Different letters (a, b, c, and d) in each column (as superscript) mean significant difference between hydrolysates (*p* < 0.05).

AA	P1	P2	P3	P4	P5
Asp	6.61 ± 0.09	6.84 ± 0.21	6.81 ± 0.08	6.71 ± 0.29	6.79 ± 0.04
Thr	2.87 ± 0.01	2.84 ± 0.03	2.73 ± 0.03	2.93 ± 0.19	2.74 ± 0.09
Ser	4.80 ± 0.13	4.92 ± 0.10	4.82 ± 0.31	4.85 ± 0.12	4.83 ± 0.04
Glu	10.44 ± 0.17	11.01 ± 0.04	10.61 ± 0.03	10.95 ± 0.09	10.40 ± 0.04
Gly	21.74 ± 0.10	21.27 ± 1.66	22.58 ± 0.12	21.49 ± 1.18	22.46 ± 0.25
Ala	9.12 ± 0.12	9.54 ± 0.38	9.43 ± 0.02	9.63 ± 0.09	9.41 ± 0.03
Cys	0.41 ± 0.23	0.25 ± 0.10	0.39 ± 0.10	0.27 ± 0.02	0.31 ± 0.02
Val	2.02 ± 0.29	1.83 ± 0.07	1.98 ± 0.05	1.80 ± 0.09	1.77 ± 0.04
Met	2.86 ± 0.34	2.46 ± 0.10	2.72 ± 0.13	2.47 ± 0.01	2.43 ± 0.02
Ile	1.51 ± 0.25	1.22 ± 0.08	1.40 ± 0.01	1.22 ± 0.01	1.32 ± 0.03
Leu	2.55 ± 0.05	2.54 ± 0.01	2.50 ± 0.01	2.69 ± 0.30	2.45 ± 0.01
Tyr	0.60 ± 0.02	0.51 ± 0.01	0.56 ± 0.09	0.53 ± 0.03	0.60 ± 0.09
Phe	2.20 ± 0.06	2.11 ± 0.13	2.25 ± 0.05	2.09 ± 0.08	2.35 ± 0.03
His	1.40 ± 0.04	1.43 ± 0.07	1.44 ± 0.07	1.42 ± 0.04	1.45 ± 0.02
Lys	3.52 ± 0.02	3.83 ± 0.30	3.92 ± 0.24	3.77 ± 0.18	3.58 ± 0.03
Arg	8.67 ± 0.16	8.50 ± 0.47	8.34 ± 0.17	8.84 ± 0.57	8.36 ± 0.11
OHPro	8.19 ± 1.11 ^a^	7.50 ± 0.17 ^a^	7.29 ± 0.25 ^a^	7.57 ± 0.18 ^a^	7.75 ± 0.21 ^a^
Pro	10.53 ± 0.07 ^a^	11.39 ± 0.22 ^b^	10.20 ± 0.09 ^c^	10.75 ± 0.31 ^ad^	11.01 ± 0.04 ^d^
Pr (%)	91.0 ± 3.5 ^a^	91.5 ± 2.7 ^a^	82.2 ± 2.9 ^b^	88.9 ± 3.3 ^a^	89.3 ± 2.6 ^a^
TE/TA (%)	27.6 ± 1.2 ^a^	26.8 ± 1.0 ^a^	26.4 ± 0.5 ^a^	27.2 ± 0.7 ^a^	26.6 ± 0.3 ^a^

**Table 5:** Molecular weight (kDa) of the distributions of gelatin from salmon shown in Figure 1. Et: elution time; *M*_w_: weight average molecular weight; *M*_n_: number average molecular weight; and PDI: polydispersity index. Peak area (%) corresponds to refractive index detector. Values are represented as the mean ± standard deviations (*n* = 2).

Procedure	Peak Number	Et (min)	*M*_w_ (kDa)	PDI	Peak Area (%)
P1	1-High *M*_w_	36.1–42.2	-	-	5.0 ± 1.1
	2	43.4 ± 0.6	346.5 ± 2.3	1.008	6.5 ± 0.6
	3	44.6 ± 0.0	214.0 ± 5.2	1.020	23.0 ± 1.7
	4	48.0 ± 0.0	119.0 ± 1.6	1.005	28.0 ± 0.1
	5-Low *M*_w_	49.1–68.3	-	-	37.5 ± 3.2
P2	1	40.6–44.2	312.1 ± 10.7	1.034	1.3 ± 0.4
	2	44.2–46.9	187.8 ± 6.8	1.018	7.2 ± 0.9
	3	48.3 ± 0.1	110.4 ± 3.2	1.008	24.3 ± 0.8
	4-Low *M*_w_	49.1–65.0	-	-	67.3 ± 6.3
P3	1	45.8–48.9	111.6 ± 28.0	1.045	6.0 ± 5.7
	2-Low *M*_w_	48.9–70.5	-	-	94.0 ± 5.7
P4	1-High *M*_w_	39.4–42.9	-	-	1.5 ± 1.0
	2	44.4 ± 0.7	328.3 ± 21.6	1.004	4.2 ± 2.9
	3	45.5 ± 0.3	212.5 ± 9.8	1.016	23.7 ± 5.5
	4	48.7 ± 0.3	119.3 ± 5.3	1.011	37.9 ± 3.7
	5-Low *M*_w_	49.8–65.0	-	-	32.7 ± 5.7
P5	1-High *M*_w_	34.2–41.1	-	-	10.4 ± 0.2
	2	41.1–42.3	482.3 ± 12.5	1.006	6.0 ± 0.5
	3	43.1 ± 0.0	351.7 ± 10.5	1.008	9.9 ± 0.8
	4	44.8 ± 0.0	221.8 ± 8.5	1.017	24.0 ± 1.2
	5	48.1 ± 0.0	121.8 ± 3.5	1.006	27.7 ± 0.1
	6-Low *M*_w_	49.4–65.1	-	-	22.0 ± 2.2

**Table 6:** Assignments to secondary structure (*β*-sheet, triple *α*-helix) for salmon gelatin.

Secundary Structure Elements	WaveNumber (cm^−1^)
*β*-Turn/*β*-Sheet	1633
Random Coil	1643, 1650
Triple *α*-Helix	1658
*β*-Turn/ *β*-Sheet	1680

**Table 7:** Mass balance, chemical, and bioactive properties of hydrolysates from salmon skin residues applying alcalase (CH1 and CH2) and papain (CH3 and CH4). Y_dig_: yield of digestion process. Y_oil_: percentage of oil recovered. Y_skin_: percentage of final solid produced (non-digestible skin). Pr: soluble protein by Lowry method. Hm: maximum hydrolysis degree from Weibull equation. *vm*: maximum rate of hydrolysis from Weibull equation. I_ACE_: maximum ACE activity. IC_50_: protein-hydrolysate concentration that generates a 50% of I_ACE_. Different letters (a, b, c, and d) in each column (as superscript) mean significant difference between hydrolysates (*p* < 0.05).

	CH1	CH2	CH3	CH4
Mass balance and hydrolysates characteristics				
Y_dig_ (%)	72.0 ± 2.5 ^a^	79.3 ± 1.6 ^b^	60.3 ± 2.1 ^c^	63.8 ± 1.1 ^d^
Y_oil_ (%)	15.8 ± 0.8 ^a^	14.0 ± 0.7 ^b^	14.6 ± 0.8 ^b^	14.3 ± 0.5 ^b^
Y_skin_ (%)	6.6 ± 0.5 ^a^	4.5 ± 0.7 ^b^	32.4 ± 1.6 ^c^	29.2 ± 0.8 ^d^
Pr (g/L)	48.1 ± 0.5 ^a^	47.6 ± 1.2 ^a^	16.2 ± 0.5 ^b^	26.6 ± 0.9 ^c^
*H_m_* (%)	14.3 ± 0.3 ^a^	20.0 ± 0.4 ^b^	4.3 ± 0.1 ^c^	6.8 ± 0.1 ^d^
*v_m_* (% min^−1^)	0.20 ± 0.03 ^a^	0.18 ± 0.01 ^a^	0.09 ± 0.01 ^c^	0.05 ± 0.01 ^d^
Amino acid composition of hydrolysates				
Gly (%)	12.1 ± 0.3 ^a^	11.0 ± 0.3 ^b^	11.0 ± 0.4 ^b^	12.2 ± 0.3 ^a^
Glu (%)	12.6 ± 0.2 ^a^	12.3 ± 0.4 ^a^	18.3 ± 0.7 ^b^	15.6 ± 0.5 ^c^
Pro (%)	7.4 ± 0.1 ^a^	6.4 ± 0.0 ^b^	9.0 ± 0.9 ^c^	7.9 ± 0.1 ^d^
OHPro (%)	3.9 ± 0.0 ^a^	3.8 ± 0.1 ^a^	6.8 ± 0.3 ^b^	5.2 ± 0.4 ^c^
TE/TA (%)	38.7 ± 0.2 ^a^	41.3 ± 0.1 ^b^	32.3 ± 1.0 ^c^	36.0 ± 1.0 ^d^
Antihypertensive and digestibility properties				
Dig (%)	82.4 ± 1.4 ^a^	85.9 ± 1.8 ^b^	74.9 ± 1.2 ^c^	77.2 ± 2.5 ^c^
I_ACE_ (%)	74.3 ± 7.1 ^a^	82.3 ± 4.3 ^a^	41.3 ± 3.5 ^b^	49.5 ± 2.6 ^c^
IC_50_ (µg Pr/mL)	189.3 ± 10.3 ^a^	71.2 ± 6.9 ^b^	732.2 ± 32.6 ^c^	562.2 ± 9.9 ^d^

**Table 8:** Molecular weight (kDa) of hydrolysates of salmon skin after gelatin extraction shown in Figure S2; *M*_w_: weight average molecular weight; *M*_n_: number average molecular weight; and PDI: polydispersity index. Values are represented as the mean ± standard deviations (*n* = 2).

Enzyme	Time	*M*_n_ (kDa)	*M*_w_ (kDa)	PDI
Alcalase	2 h	1157 ± 156	2068 ± 49	1.788
	4 h	883 ± 121	1810 ± 44	1.938
Papain	2 h	2749 ± 137	8054 ± 137	2.930
	4 h	2721 ± 209	7567 ± 454	2.781

## Data Availability

Not applicable.

## Supplementary Materials

The following are available online at https://www.mdpi.com/article/10.3390/polym13162828/s1, Figure S1: Flowcharts summarizing the protocols studied for the production of gelatins from salmon skin by-products. Chemical treatments in P1 and P2 were run at 22 °C and 4 °C, respectively. Chemical treatments in P4 and P5 were run at 22 °C and 4 °C, respectively; Figure S2. GPC eluograms of hydrolysates of salmon skin after gelatin extraction. (a) CH1: alcalase 2 h; (b) alcalase 4 h; (c) papain 2 h; (d) papain 4 h. Blue line: right angle light scattering; red line: refractive index; black line: ultraviolet (232 nm). Table S1: Values obtained from the analysis of the TGA and DTGA curves of salmon gelatin P1; Table S2: Values obtained from the analysis of DSC of salmon gelatin P1.
