# Supplementary material for: Characterization of Gelatin and Hydrolysates from Valorization of Farmed Salmon Skin By-Products
Source: Polymers (Basel). 2021 Aug 23;13(16):2828. doi: 10.3390/polym13162828 (PMC8398820; doi:10.3390/polym13162828)
Supplement: Supplementary file 1 [file polymers-13-02828-s001.zip › polymers-1337719-supplementary.pdf]

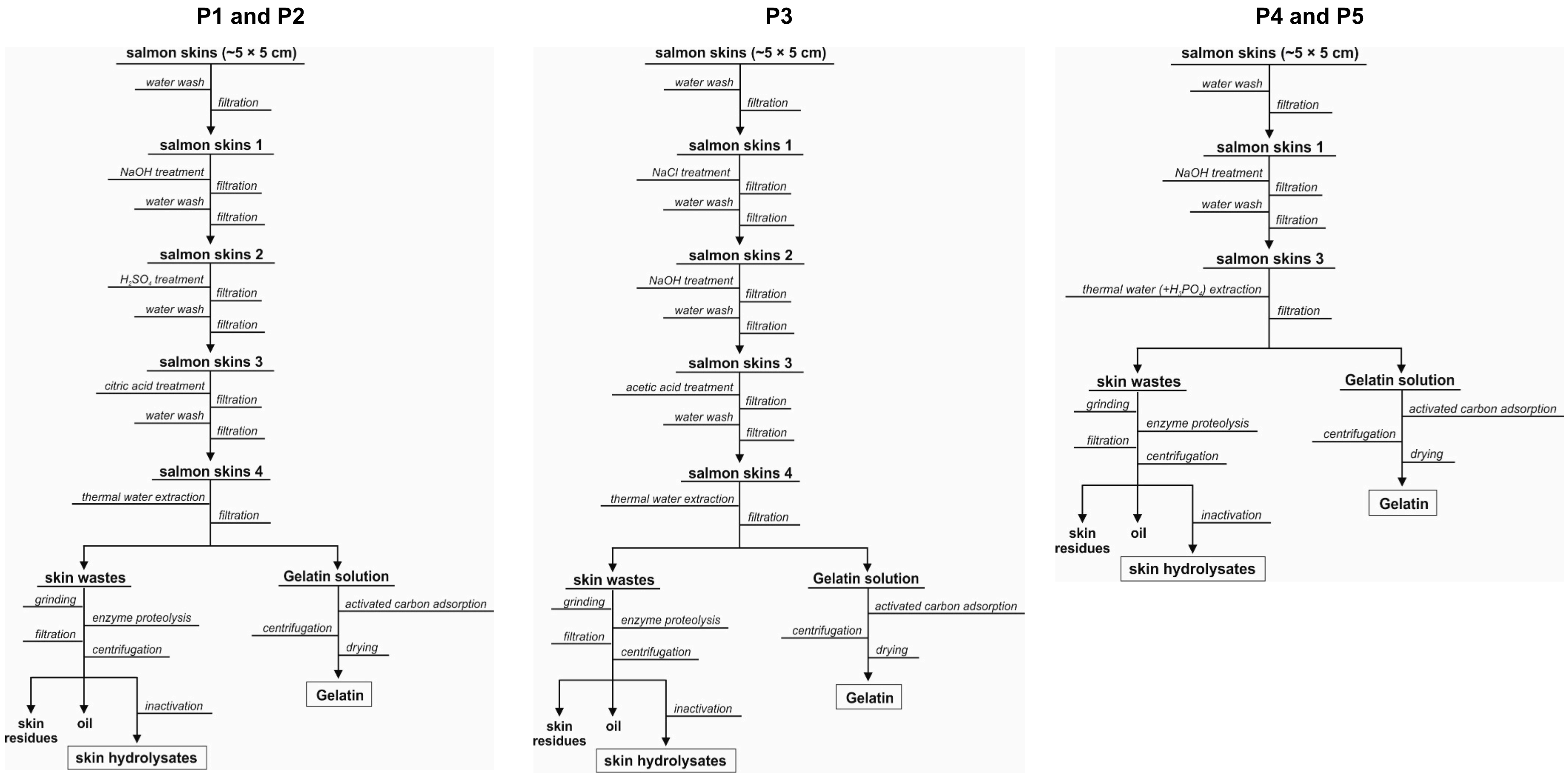

Figure S1. Flowcharts summarizing the protocols studied for the production of gelatins from salmon skin by-products. Chemical treatments in P1 and P2 were run at 22ºC and 4ºC, respectively. Chemical treatments in P4 and P5 were run at 22ºC and 4ºC, respectively.

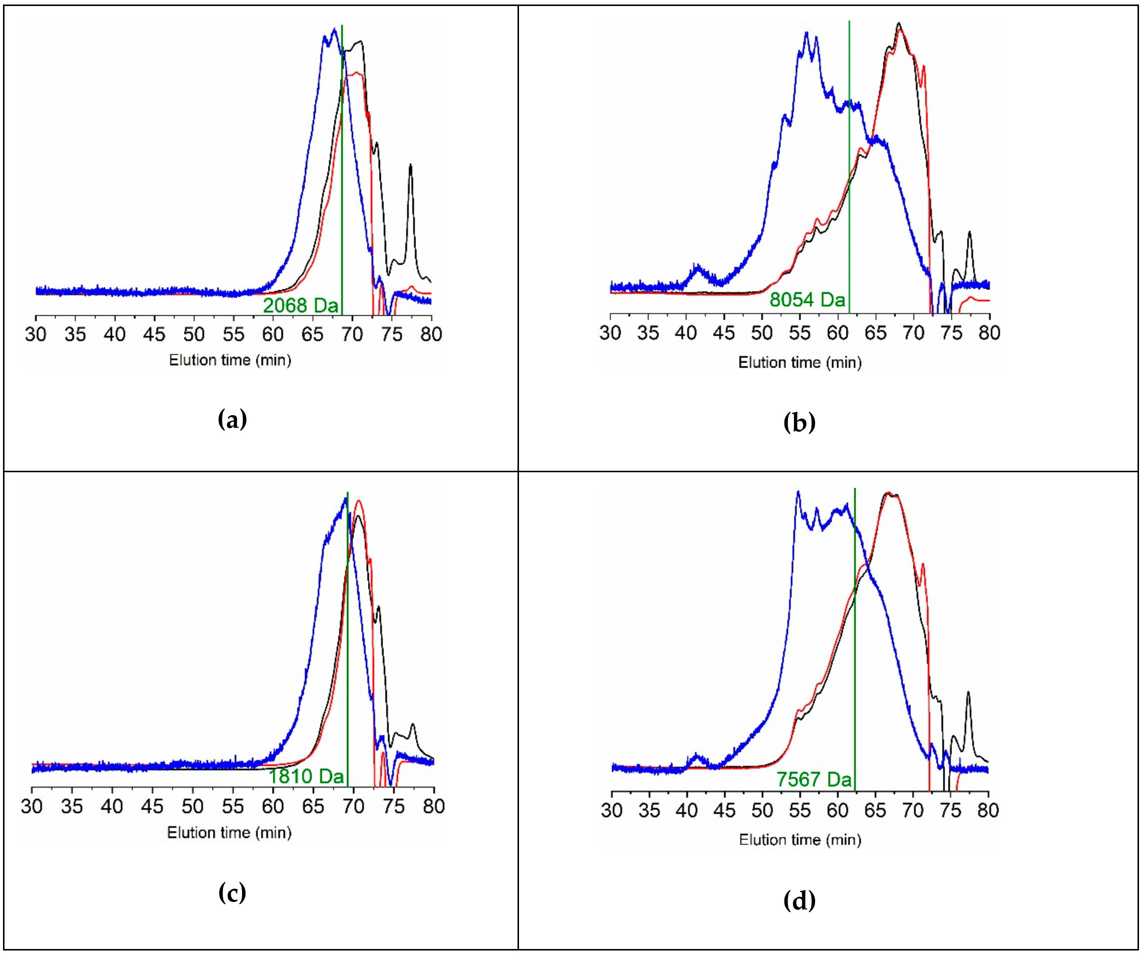

Figure S2. GPC eluograms of hydrolysates of salmon skin after gelatine extraction. **(a)** CH1: alcalase 2h; **(b)** alcalase 4h; (**c**) papain 2h; (**d**) papain 4h. Blue line: right angle light scattering; red line: refractive index; black line: ultraviolet (232 nm).

Table S1. Values obtained from TGA and DTGA analysis of salmon gelatin P1. Onset temperatures, T_{onset}; maximum temperatures, T_{max}; and *Weight loss (%)* at 5 °C/min.

**Steps**	**T Onset (°C)**	T_{max} (°C)	*Weight loss (%)*
**1**	39.6	103	-7.6
**2**	252	303	-62.8

Table S2. Values obtained from the analysis of DSC of salmon gelatin P1. Onset temperatures, T_{onset}; maximum temperatures, T_{max}; and enthalpy, ΔH, at 5 °C/min.

**Point**	**T Onset (°C)**	T_{max} (°C)	**ΔH (J/g)**
**1**	20.6	86.5	75.94
**2**	233.8	292.5	286.97
